# Supplementary material for: Observation of electronic modes in open cavity resonator
Source: Nat Commun. 2023 Jan 26;14:415. doi: 10.1038/s41467-023-36012-2 (PMC9876930; doi:10.1038/s41467-023-36012-2)
Supplement: Supplementary file 1 — Supplementary Information [file 41467_2023_36012_MOESM1_ESM.pdf]

# Supplementary Information for
# "Observation of Electronic Modes in Open Cavity Resonator"

Hwanchul Jung[1,2*], Dongsung T. Park[3*], Seokyeong Lee[3], Uhjin Kim[4], Chanuk Yang[4], Jehyun Kim[5], V. Umansky[6], Dohun Kim[5], H.-S. Sim[3], Yunchul Chung[1,2†,‡], Hyoungsoon Choi[3†,§], Hyung Kook Choi[4†,#]

[1] *Department of Physics, Pusan National University, Busan 46241, Republic of Korea.*

[2] *Quantum Matter Core-Facility, Department of Physics, Pusan National University, Busan 46241, Republic of Korea.*

[3] *Department of Physics, KAIST, Daejeon 34141, Republic of Korea.*

[4] *Department of Physics, Research Institute of Physics and Chemistry, Jeonbuk National University, Jeonju 54896, Republic of Korea.*

[5] *Department of Physics and Astronomy, and Institute of Applied Physics, Seoul National University, Seoul 08826, Korea.*

[6] *Department of Condensed Matter Physics, Weizmann Institute of Science, Rehovot 76100, Israel.*

[*] These authors contributed equally: H. J. and D. T. P.

[†] These authors jointly supervised the work: Y. C., H. C., H. K. C.

[‡] ycchung@pusan.ac.kr

[§] h.choi@kaist.ac.kr

[#] hkchoi@jbnu.ac.kr

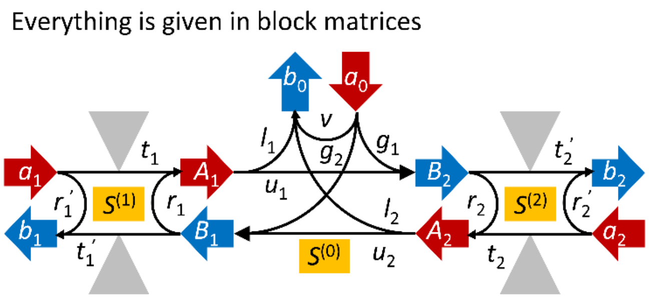


**Fig. S1 Open cavity resonator model.** Incoming and outgoing modes to the scattering centers, i.e. mirror QPCs, have been indicated with $a_i$ ($A_i$) and $b_i$ ($B_i$), respectively. The measurement lead modes have been written in lowercase, and the cavity resonator modes have been written in uppercase. The intra-cavity scattering and the coupling to the open sides are described within $S^{(0)}$.

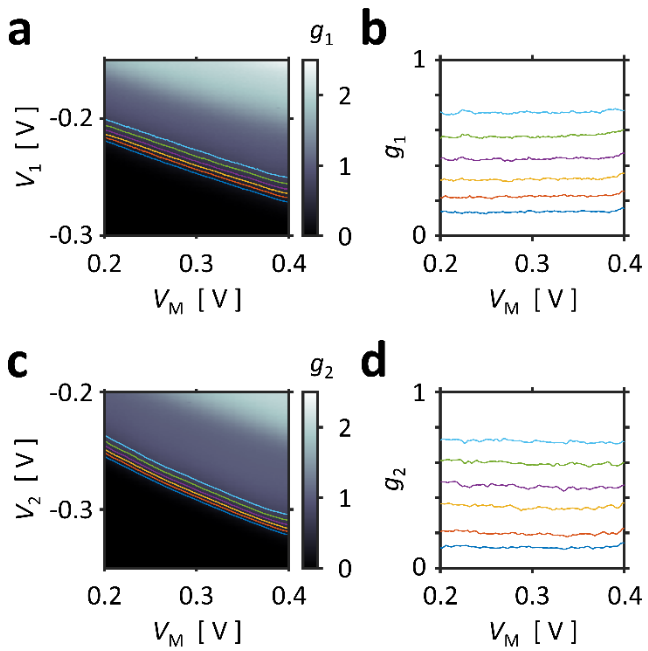


**Fig. S2 QPC tuning.** **a** QPC conductance $g_1$ for mirror 1u-1d plotted as a function of the modulation gate voltage $V_\mathrm{M}$ and the mirror gate voltages $V_1$. Note that the mirror gates have been tuned identically, i.e. $V_{1\mathrm{u}} = V_{1\mathrm{d}} = V_1$. Since $V_\mathrm{M}$ affects $g_1$, $V_1$ has to be adjusted to maintain a constant mirror transmission. The condition for constant conductance has been found by interpolation and drawn onto the $V_1$-$V_\mathrm{M}$-$g_1$ image. **b** Using the interpolated data, $g_1$ has been measured against $V_\mathrm{M}$ with implicit adjustments to $V_1$. We see that $g_1$ is maintained nearly constant as desired. **c**, **d** Similar analysis for the mirror 2u-2d.

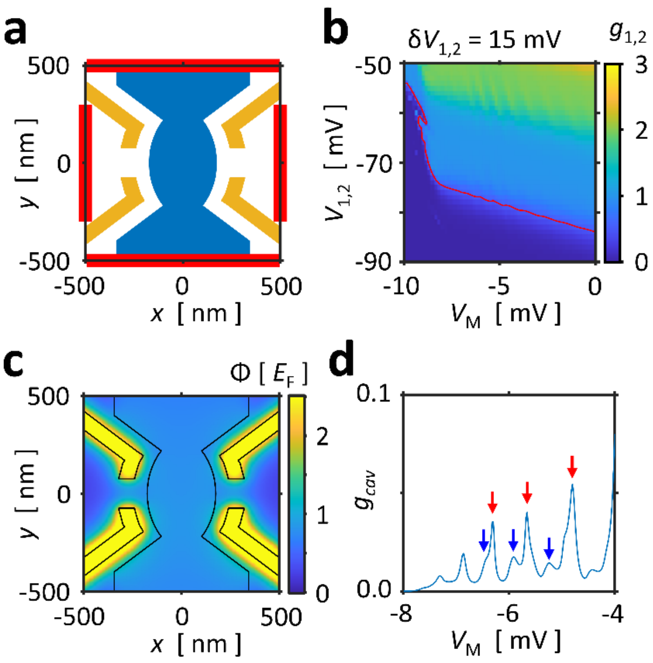


**Fig. S3 KWANT simulation parameters. a** Schematic illustration of the simulated device using the lithographic dimensions of the experimental sample. Mirror gates have been shaded in yellow, modulation gate in blue, and the lead positions in thick red borders. **b** Constant conductance lines extracted similar to Fig. S1a. The detuning voltage has been applied a priori, e.g. $V_{1\mathrm{u}} = V_1 + \delta V_1/2$ and $V_{1\mathrm{d}} = V_1 - \delta V_1/2$ where $V_1 = V_1(V_\mathrm{M}; g_1)$ and $\delta V_1 = V_{1\mathrm{u}} - V_{1\mathrm{d}} = 15\,\mathrm{mV}$. Note that the simulated device is symmetric with respect to reflection across $x = 0$. Only the mean voltage $V_1 = V_2$ has been labelled. Red line traces the conditions for $g_{1,2} = 0.5$. **c** Electrostatic potential $\phi$ applied by the gates at the conditions for Fig. 5f. The gates boundaries have been delineated with black lines, and the colorbar has been given in units of the Fermi energy $E_\mathrm{F} = 8.2\,\mathrm{meV}$. **d** The $g_\mathrm{cav}$ calculated along the red line in **b**, corresponding to $B = 0$ in Fig. 5e.

**Supplementary Note 1: Landauer-Büttiker Model of a Cavity Resonator**

In similar spirit to optical systems[1], the open cavity resonator can be treated in the Landauer-Büttiker formalism via scattering matrices. An open cavity has three types of measurement leads: two corresponding to the electron reservoirs beyond the cavity mirrors, and one corresponding to the open sides. Three S-matrices are defined in this picture: $S^{(1,2)}$ describing the tunneling across the cavity mirror (indexed $i = 1,2$) and $S^{(0)}$ describing the intra-cavity reflections and diffractive loss to the open sides. See Fig. S1 for the overview of the model. Note that the variables in the following discussion is either in matrix or block matrix form, and all amplitudes are non-scalar entities for generality. However, the formulas derived here have been applied to the main text in scalar form for simplicity, i.e. $t_1$, $r_1$, $u_1$, and so forth in Eq. 1 are scalars.

The S-matrix of the cavity mirror $i$ is written as

$$S^{(i)}: \begin{pmatrix} a_i \\ B_i \end{pmatrix} \rightarrow \begin{pmatrix} b_i \\ A_i \end{pmatrix} = \begin{pmatrix} r_i' & t_i' \\ t_i & r_i \end{pmatrix} \begin{pmatrix} a_i \\ B_i \end{pmatrix} \tag{S1}$$

where $a_i$ ($B_i$) are the column matrices of amplitudes for modes heading towards the cavity mirror from the lead (cavity) side, $b_i$ ($A_i$) those heading away from the cavity mirror towards the lead (cavity) side, and $r_i$ or $t_i$ ($r_i'$ or $t_i'$) blocks of the $S^{(i)}$ matrix corresponding to the reflection or tunneling amplitudes of the mirrors heading into (out of) the cavity. By rearranging $a_{12} = (a_1; a_2)$ and $b_{12} = (b_1; b_2)$, where the semicolon indicates a row change instead of a column change, we may rewrite this in the succinct form

$$S^{(12)}: \begin{pmatrix} B \\ a_{12} \end{pmatrix} \rightarrow \begin{pmatrix} A \\ b_{12} \end{pmatrix} = \begin{pmatrix} r & t \\ t & r' \end{pmatrix} \begin{pmatrix} B \\ a_{12} \end{pmatrix} \tag{S2}$$

which describes all the scatterings across the cavity mirrors. This notation makes it simpler to work with the intra-cavity scattering and cavity-side coupling, given by

$$S^{(0)}: \begin{pmatrix} a_0 \\ A \end{pmatrix} \rightarrow \begin{pmatrix} b_0 \\ B \end{pmatrix} = \begin{pmatrix} v & l \\ g & u \end{pmatrix} \begin{pmatrix} a_0 \\ A \end{pmatrix} \tag{S3}$$

where $a_0$ ($b_0$) is the amplitude matrix of modes from the open cavity sides heading into (out of) the cavity, $g$ and $v$ the scattering amplitude matrix from the incoming side modes to into the cavity or back to the sides, $l$ the amplitude matrix of diffraction loss from the cavity to the open reservoirs, and $u$ the transmission amplitude matrix from one side of the cavity mirror to the other. A few small notes. Both sides of the open cavity are incorporated in $a_0$ and $b_0$: the upper/lower parts of the open sides can be resolved by splitting the basis, e.g. $a_0 \rightarrow \left(a_0^{(\mathrm{up})}; a_0^{(\mathrm{down})}\right)$, but we have not done so here as it does not add to the discussion. Also, all these matrices are unitary by construction, and the diffraction 'loss' term $l$ is lossy in the sense that electrons exit the cavity

through the sides. Specifically, we note that $\|u\|^2 + \|l\|^2 = \dim(u)$ where $\|u\|^2 = \mathrm{Tr}(u^\dagger u)$. In a lossless cavity, $\|l\|^2 = 0$ and all the eigenvalues of $u$ are in the simple form $\exp(i\theta)$ as in the usual Fabry-Perot model. The total cavity S-matrix we wish to find can be algebraically solved for[2,3]:

$$S\colon \begin{pmatrix} a_0 \\ a_{12} \end{pmatrix} \to \begin{pmatrix} b_0 \\ b_{12} \end{pmatrix} = \begin{pmatrix} v + lr[I_c - ur]^{-1}g & l[I_c - ru]^{-1}t \\ t'[I_c - ur]^{-1}g & r' + t'[I_c - ur]^{-1}ut \end{pmatrix} \begin{pmatrix} a_0 \\ a_{12} \end{pmatrix} \tag{S4}$$

where $I_c$ is the identity matrix with $\dim(I_c) = \dim(A) = \dim(B)$ and we recognize that $[I_c - ur]^{-1}$, or nearly equivalently $[I_c - ru]^{-1}$, corresponds to the enhancement factor described in optical systems which gives the Fabry-Perot spectrum its signature lineshape[4]. The enhancement factor is present in every term, in agreement with optical systems where all transmission from a Fabry-Perot cavity is proportional to the said factor[4]. Note that $[I_c - ur]^{-1}u = u[I_c - ru]^{-1}$ and $r[I_c - ur]^{-1} = [I_c - ru]^{-1}r$ for invertible[5] $r$ and $u$.

For cavity resonances, we are usually interested in $a_{12} \to b_{12}$ and $a_{1(2)} \to b_{2(1)}$ in particular. Here, we assume that there is no additional scattering within the cavity, i.e. a rightwards propagating wave does not reflect leftwards before hitting the mirror. This assumption is mathematically expressed as $u = \mathrm{diag}(u_1, u_2)$, and the full form of $S\colon (a_1; a_2; a_0) \to (b_1; b_2; b_0)$ is written out below for reference:

$$S = \begin{pmatrix} r_1' + t_1'u_2r_2\dfrac{1}{I_2 - u_1r_1u_2r_2}u_1t_1 & t_1'\dfrac{1}{I_1 - u_2r_2u_1r_1}u_2t_2 & t_1'\dfrac{1}{I_1 - u_2r_2u_1r_1}(g_2 + u_2r_2g_1) \\ t_2'\dfrac{1}{I_2 - u_1r_1u_2r_2}u_1t_1 & r_2' + t_2'u_1r_1\dfrac{1}{I_1 - u_2r_2u_1r_1}u_2t_2 & t_2'\dfrac{1}{I_2 - u_1r_1u_2r_2}(u_1r_1g_2 + g_1) \\ (l_1 + l_2r_2u_1)\dfrac{1}{I_1 - r_1u_2r_2u_1}t_1 & (l_1r_1u_2 + l_2)\dfrac{1}{I_2 - r_2u_1r_1u_2}t_2 & V \end{pmatrix}$$

where $I_i$ is the identity matrix with $\dim(I_i) = \dim(A_i) = \dim(B_i)$ and

$$\begin{aligned} V &= v + l_1r_1[I_1 - u_2r_2u_1r_1]^{-1}(g_2 + u_2r_2g_1) + l_2r_2[I_2 - u_1r_1u_2r_2]^{-1}(u_1r_1g_2 + g_1) \\ &= v + (l_1r_1 + l_2r_2u_1r_1)[I_1 - u_2r_2u_1r_1]^{-1}g_2 + (l_1r_1u_2r_2 + l_2r_2)[I_2 - u_1r_1u_2r_2]^{-1}g_1. \end{aligned}$$

From the matrix, we can read off $t_{12} = \partial b_1/\partial a_2 = S_{12}$ as

$$t_{12} = t_1'\frac{1}{I_1 - u_2r_2u_1r_1}u_2t_2. \tag{S5}$$

and $t_{21}$ trivially given by switching $(1 \leftrightarrow 2)$. Also, we see that the diffraction losses $S_{3i}$ have terms $[I_1 - r_1u_2r_2u_1]^{-1}$ and $[I_2 - r_2u_1r_1u_2]^{-1}$. Since $t_{12} = t_1'[I_1 - u_2r_2u_1r_1]^{-1}u_2t_2 = t_1'u_2[I_2 - r_2u_1r_1u_2]^{-1}t_2$ and $t_{21}$ contain the same terms, diffraction loss current shares the same general lineshape with the cavity transmission through the mirrors.

**Supplementary Note 2: Estimating Finesse**

In an optical cavity[4], the finesse is defined as the $\mathcal{F} = \nu_{\mathrm{FSR}}/\nu_{\mathrm{c}}$ where $\nu_{\mathrm{FSR}} = 1/\tau_{\mathrm{RT}}$ is the free spectral range, given by the inverse roundtrip time, and $\nu_{\mathrm{c}} = 1/\tau_{\mathrm{c}}$ is the decay rate. Heuristically, we replaced the role of frequency with the wavenumber, i.e. $\mathcal{F}' = k_{\mathrm{FSR}}/k_{\mathrm{c}}$ where $k_{\mathrm{FSR}} = 1/2L$ is the inverse roundtrip length and $k_{\mathrm{c}} = 1/l_{\mathrm{c}}$ is the inverse decay length. The ballistic length of our 2DEG is estimated by the mean free path: $l_{\mathrm{b}} = v_{\mathrm{F}}\tau \approx 28$ µm where $v_{\mathrm{F}}$ is the Fermi velocity and $\tau = (m^*/e) \times \mu$ the mean free time given by the effective mass $m^*$, electron charge $e$, and mobility $\mu$. The coherence length is upper limited by the thermal length $l_{\mathrm{t}} = hv_{\mathrm{F}}/k_{\mathrm{B}}T_{\mathrm{e}}$ where $h$ is the Planck constant, $k_{\mathrm{B}}$ the Boltzmann constant, and $T_{\mathrm{e}}$ the electron temperature. The electrons are limited by impurity scattering when $l_{\mathrm{t}} > l_{\mathrm{b}}$, achievable by lowering the electron temperature down to $< 30$ mK, and the decay length is simply $l_{\mathrm{c}} \approx l_{\mathrm{b}}$. For a cavity with size $L = 0.5$ µm using our 2DEG sample, the finesse for a mode with zero diffractive losses is upper limited by $\mathcal{F}' < \pi l_{\mathrm{c}}/L \approx 176$, which corresponds to a loss rate of ≈ 3.5 % per roundtrip.

## Supplementary References


1. Stone, M., Suleymanzade, A., Taneja, L., Schuster, D. I. & Simon, J. Optical mode conversion in coupled Fabry–Perot resonators. *Opt. Lett.* **46**, 21–24 (2021).
2. Datta, S. Transmission function, S-matrix and Green's functions. in *Electronic Transport in Mesoscopic Systems* 117–174 (Cambridge University Press, 1995). doi:DOI: 10.1017/CBO9780511805776.004
3. Heikkila, T. T. *The Physics of Nanoelectronics: Transport and Fluctuation Phenomena at Low Temperatures*. (Oxford University Press, 2013).
4. Ismail, N., Kores, C. C., Geskus, D. & Pollnau, M. Fabry-Perot resonator: spectral line shapes, generic and related Airy distributions, linewidths, finesses, and performance at low or frequency-dependent reflectivity. *Opt. Express* **24**, 16366–16389 (2016).
5. Lu, T.-T. & Shiou, S.-H. Inverses of 2 × 2 block matrices. *Comput. Math. with Appl.* **43**, 119–129 (2002).
