# Supplementary material for: Observation of electronic modes in open cavity resonator
Source: Nat Commun. 2023 Jan 26;14:415. doi: 10.1038/s41467-023-36012-2 (PMC9876930; doi:10.1038/s41467-023-36012-2)
Supplement: Supplementary file 3 — Description of Additional Supplementary Files [file 41467_2023_36012_MOESM3_ESM.pdf]

File name: Supplementary Software 1

Description:

1. Overview

This document explains how to run the source code used in this study.

2. File structure

This source code contains necessary functions and key data to redo the simulations.

The “Calculation” folder contains the functions which defines the scattering center (`mesa.py`) and gates (`gate.py`). Also, it contains a simulation managing function (`plan.py`) and the actual simulation calculating files (`result4.py, all_scan.py, and all_scan_fixed.py`)

In the “data” folder, we put crucial data to run our simulations. Usually, some 2D scans might take hours to finish. Therefore, in order to reduce the running time of the all the source code, we put the data which is necessary to proceed all the simulation.

“`utill.py`” in the main folder provides the functions which easily load the previous data.

The main code is “`Source code for publication.ipynb`”. It contains all the necessary code to recreate the simulation results.

Lastly, in the main folder, “`confocal_sym.mat`” has all the coordinates which defines the gates, “`paper.rc`” defines the style of the plotting, and “`log.xlsx`” contains the empty log to run the simulation properly. “IMG1.png” and “IMG2.png” are images inside a main source code.

3. Installation requirements

```
1.  pip install -r requirements.txt
```

There are some necessary packages for running this code. Basically, almost all the libraries except KWANT are initially installed when someone install a full Anaconda distribution. However, for a new virtual environment or mini conda distribution, some of necessary packages may not be installed. Therefore, use this code on python to install necessary requirements.

4. Run Jupyter notebook and the simulation

The source code is written in Jupyter notebook file (.ipynb). Therefore, to read the source code, the Jupyter notebook is necessary. To run the Jupyter notebook, type below on python.

```
1.  jupyter notebook
```

If Jupyter notebook is not available, then again install Jupyter notebook using pip as

```
1.  pip install notebook
```

Open “`Source code for publication.ipynb`” on the Jupyter notebook and run the code in order.
