# Supplementary figures and images for: Observation of electronic modes in open cavity resonator
Source: Nat Commun. 2023 Jan 26;14:415. doi: 10.1038/s41467-023-36012-2 (PMC9876930; doi:10.1038/s41467-023-36012-2)

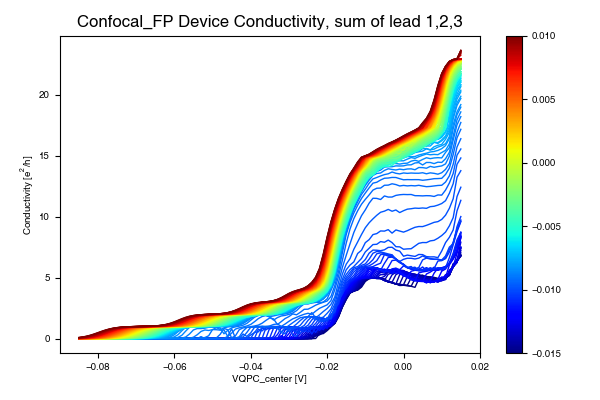

Supplement: Supplementary file 4 — Supplementary Software 1 [file 41467_2023_36012_MOESM4_ESM.zip › data/Confocal_FP all_scan 220304-1604-191693950/figures/1d scan.png]

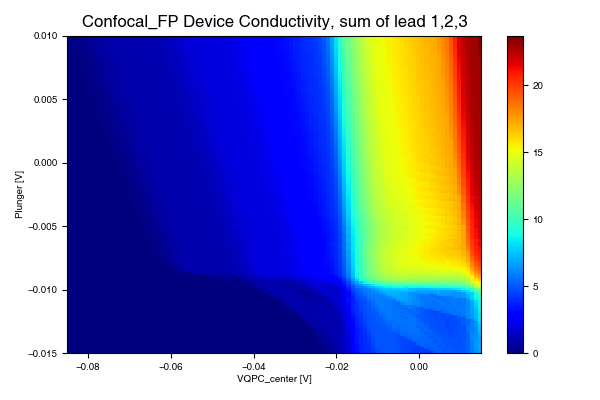

Supplement: Supplementary file 4 — Supplementary Software 1 [file 41467_2023_36012_MOESM4_ESM.zip › data/Confocal_FP all_scan 220304-1604-191693950/figures/2d scan.png]

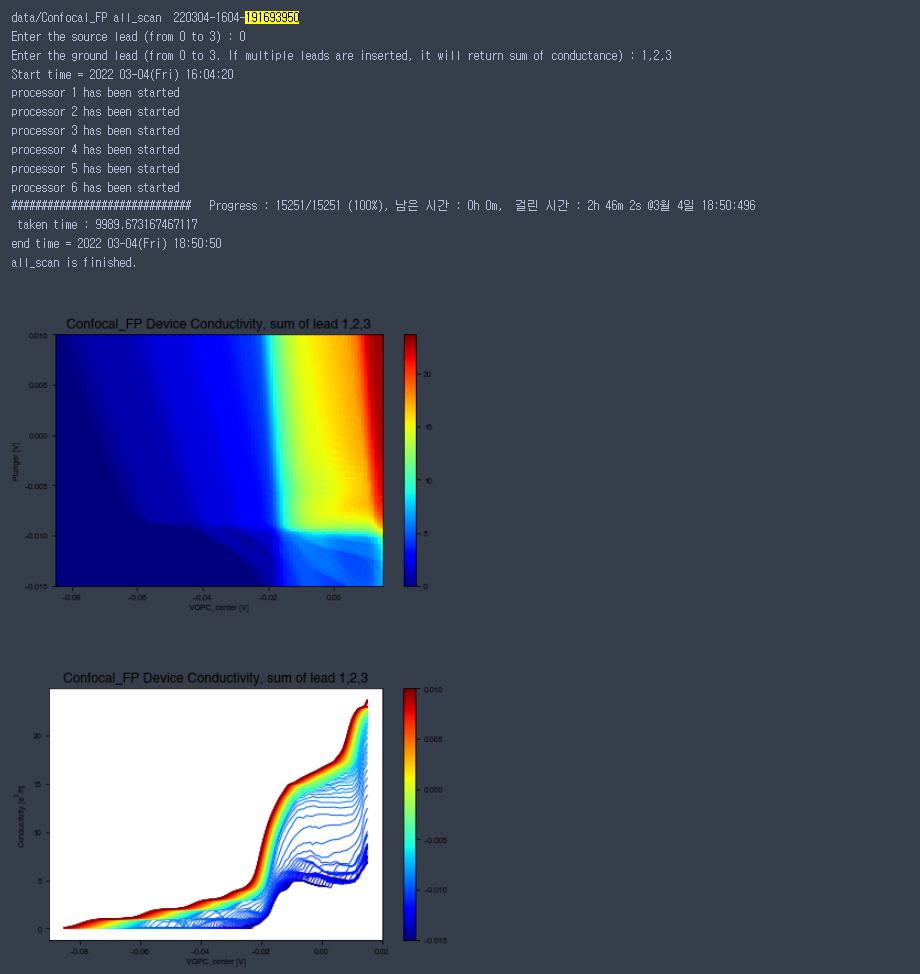

Supplement: Supplementary file 4 — Supplementary Software 1 [file 41467_2023_36012_MOESM4_ESM.zip › IMG1.png]

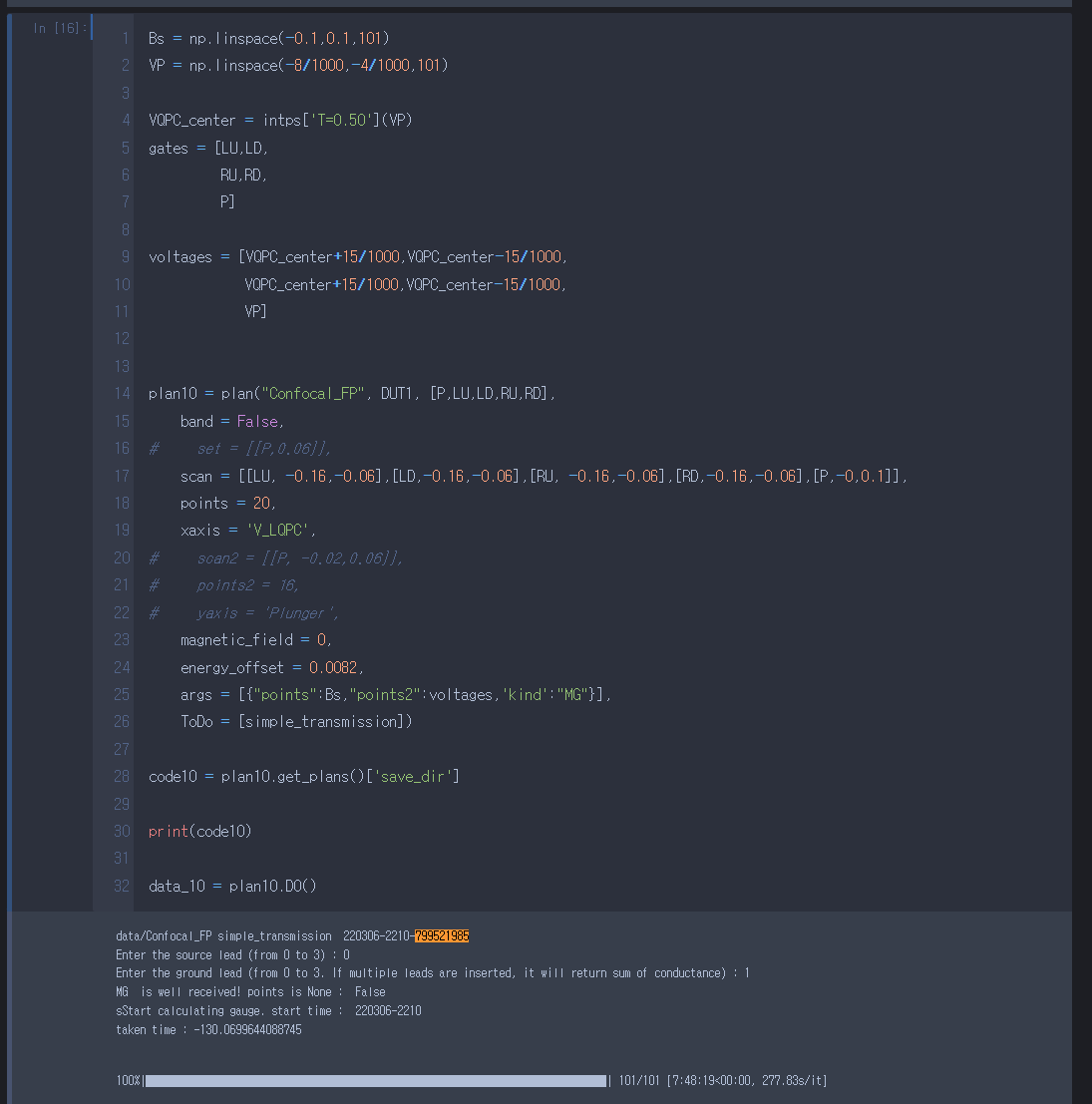

Supplement: Supplementary file 4 — Supplementary Software 1 [file 41467_2023_36012_MOESM4_ESM.zip › IMG2.png]
